# Supplementary material for: Liquid–liquid phase separation within fibrillar networks
Source: Nat Commun. 2023 Sep 29;14:6085. doi: 10.1038/s41467-023-41528-8 (PMC10539382; doi:10.1038/s41467-023-41528-8)
Supplement: Supplementary file 1 — Supplementary Information [file 41467_2023_41528_MOESM1_ESM.pdf]

Supplementary Information

Liquid–liquid phase separation within fibrillar networks

Jason X. Liu[1,2], Mikko P. Haataja[1,2], Andrej Košmrlj[1,2], Sujit S. Datta[3], Craig B. Arnold[1,2], Rodney D. Priestley[2,3]*

[1]Department of Mechanical and Aerospace Engineering, Princeton University; Princeton, NJ 08544, USA.
[2]Princeton Materials Institute, Princeton University; Princeton, NJ 08544, USA.
[3]Department of Chemical and Biological Engineering, Princeton University; Princeton, NJ 08544, USA.

Correspondence to: rpriestl@princeton.edu

Contents:

Supplementary Notes 1 to 9
Supplementary Figures 1 to 22
Supplementary References

Supplementary Note 1: The solvent-exchange process

In Supplementary Fig. 1, we present the ethanol-water-decane ternary phase diagram, generated from mass fraction phase equilibria data[1,2]. The red curve indicates the binodal separating the 1-phase region (white) in which ethanol, water, and decane are miscible from the 2-phase region (grey) in which phase separation into decane-rich and decane-poor phases occurs.

At the top of the ternary phase diagram, the green circle indicates the starting system composition prior to phase separation. This composition has mass fractions w_{dec} = 0.035, w_{EtOH} = 0.965, and w_{H_2O} = 0, corresponding to 4% v/v decane in ethanol. The green line connecting the starting composition to the green circle in the bottom left corner represents the solvent exchange process in which ethanol is diffusively replaced with water, reaching a final composition of w_{dec} = 0.035, w_{EtOH} = 0, and w_{H_2O} = 0.965. Note that for these calculations, we neglect the volume of residual solvent within the gel during the solvent exchange process (refer to Methods). When the system composition intersects the red binodal line during solvent exchange (purple circle), phase separation occurs. This occurs at w_{dec} = 0.035, w_{EtOH} = 0.833, and w_{H_2O} = 0.132.

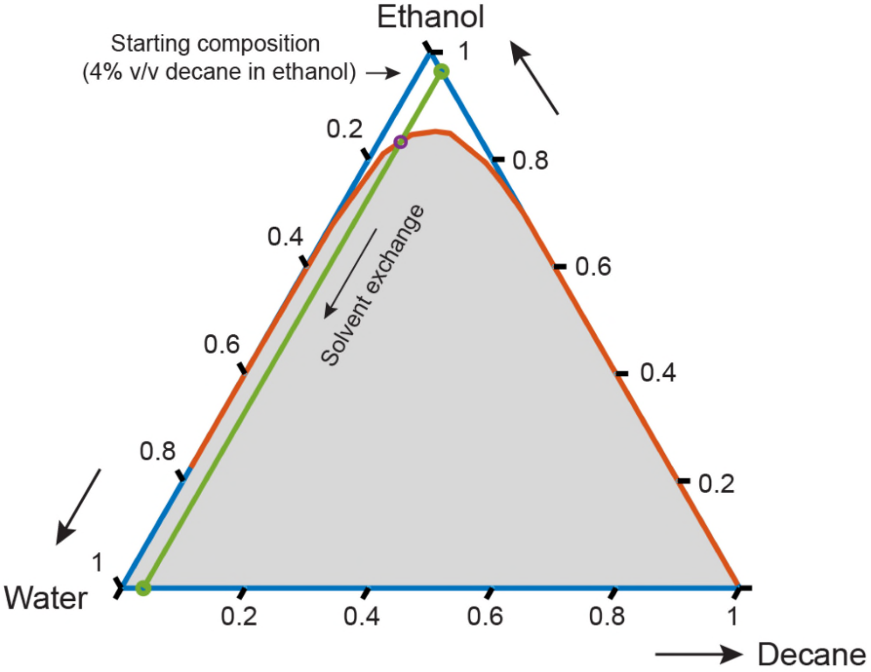

Supplementary Fig. 1: Ternary phase diagram of ethanol, water, and decane at 293 K. The white region is a 1-phase mixed region while the grey region is a 2-phase demixed region. These regions are separated by the red binodal curve. The green circle at the top of the diagram corresponds to an initial composition of 4% v/v decane in ethanol. The green line represents the solvent exchange process in which ethanol is replaced by water. The purple circle marks the intersection of the green line with the binodal curve.

In these experiments, solvent exchange occurs via diffusion of ethanol into the agarose network and diffusion of water out of the network. Experimentally, the agarose gels fill a recessed well which is 1 mm deep, and microscopy visualization is performed at the bottom of the 1 mm thick gels. Water is introduced to the top of the gel, as shown in Supplementary Fig. 2.

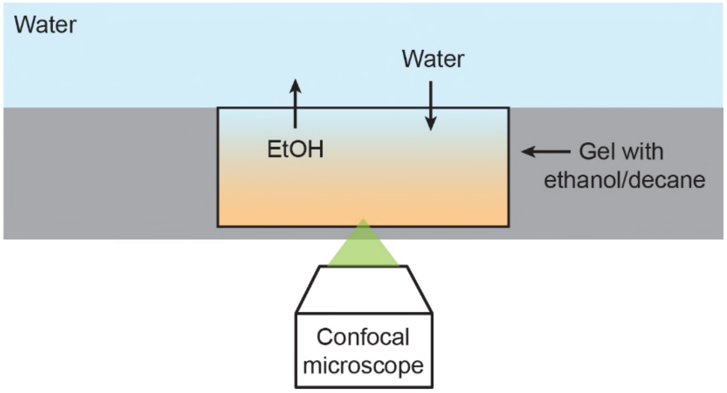

Supplementary Fig. 2: Schematic of the experimental geometry. The agarose gel is 1 mm thick and is located in a recessed well. Water is introduced to the top surface of the gel and diffuses into the gel, while ethanol diffuses out of the gel. Confocal microscopy visualization is performed at the bottom of the gel. The light blue color represents water while the tan color represents ethanol.

Here, we use a simple Fickian diffusion model[3] to estimate the water content at the bottom of the gel as a function of time.

$$C_{1\,\mathrm{mm}}(t) = C_{top} \cdot \left(1 - \mathrm{erf}\left(\frac{(1\ \mathrm{mm})}{2\sqrt{Dt}}\right)\right)$$

We approximate that the upper water reservoir has a fixed composition w_{H_2O} = 1 and that the diffusion coefficient of water into the gel is $D = 1 \times 10^{-9}$ m^2/s. Note that these are approximations and that this calculation is performed for illustrative purposes to demonstrate the kinetics of the solvent exchange process. In Supplementary Fig. 3, we plot the time evolution of the water content at the bottom of a 1 mm thick gel as a function of time. As shown in Supplementary Fig. 1, phase separation of an oil-rich and oil-poor phase occurs at w_{dec} = 0.035, w_{EtOH} = 0.833, and w_{H_2O} = 0.132. This water composition is indicated in Supplementary Fig. 3 with the purple circle and horizontal line.

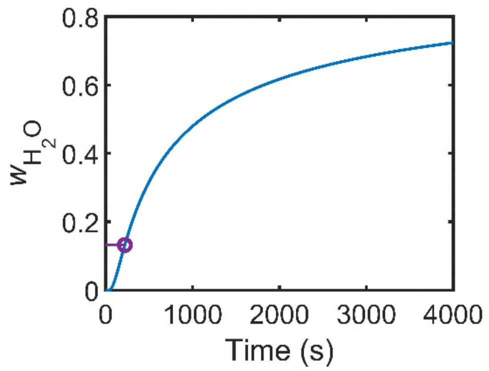

Supplementary Fig. 3: Fickian diffusion calculation which estimates the time evolution of the water content at the bottom of the gel where microscopy visualization is performed. The purple circle and horizontal line indicates a water composition of $\boldsymbol{w_{H_2O}}$ = 0.132, where phase separation is predicted to occur, from Supplementary Fig. 1.

As solvent exchange proceeds, w_{H_2O} continually rises. This rise in water content increases the interfacial tension between the decane-rich and decane-poor phases, eventually leading to the deformation of the agarose network as demonstrated in the main text. In Supplementary Fig. 4 we show measurements of the interfacial tension between a decane droplet and a water-ethanol mixture as a function of water mass fraction w_{H_2O}. In Supplementary Fig. 5 we plot an estimate of the interfacial tension as a function of time, during solvent exchange.

Note that in Supplementary Fig. 4 and Supplementary Fig. 5, $\gamma_{ow} \approx 8$ mN/m when phase separation first occurs, at w_{H_2O} = 0.132. If one refers to Fig. 6a from the main text, one can see that $\gamma_{ow} \approx 8$ mN/m lies well within the region of the blue dots for c_{gel} = 0.8%, 1.3%, and 2.0% w/w, meaning that γ_{ow} is yet insufficient to fracture the network. For c_{gel} = 0.3% w/w, $\gamma_{ow} \approx 8$ mN/m lies between the blue and red dot, meaning that interfacial tension is at the cusp of deforming or fracturing the network. This corroborates the experimental results for c_{gel} = 0.3% w/w in which we see substantial network deformation coupled with condensate growth.

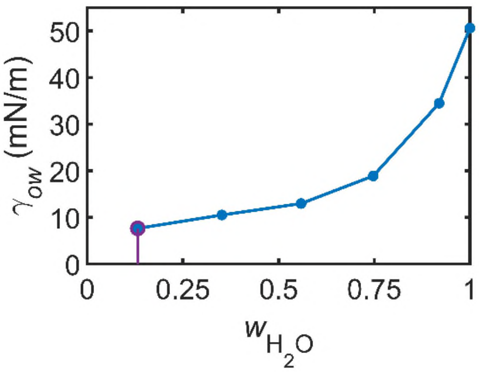

Supplementary Fig. 4: Interfacial tension between decane and a water-ethanol mixture, $\boldsymbol{\gamma_{ow}}$, with varying water concentration. The purple circle and horizontal line indicates a water composition of $\boldsymbol{w_{H_2O}}$ = 0.132, where phase separation is predicted to occur, from Supplementary Fig. 1.

Interfacial tension is measured with pendant droplet tensiometry. In these interfacial tension measurements, we first equilibrate decane with the water-ethanol mixture by vortex mixing a small volume of decane with a larger volume of the water-ethanol mixture used for a given water concentration.

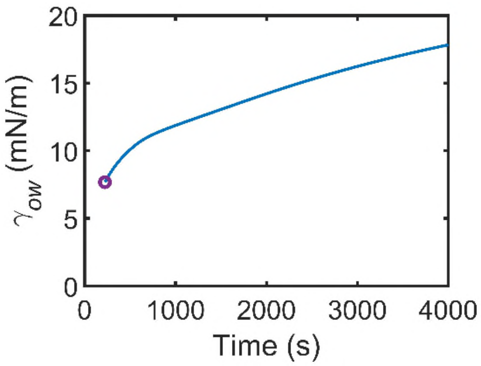

Supplementary Fig. 5: Interfacial tension between decane and a water-ethanol mixture, $\boldsymbol{\gamma_{ow}}$, vs. time as solvent exchange proceeds. Here, $\boldsymbol{\gamma_{ow}}$ is determined by reading out $\boldsymbol{\gamma_{ow}(w_{H_2O}(t))}$ from Supplementary Fig. 3 and Supplementary Fig. 4.

Supplementary Note 2: Agarose gel mesh sizes

We tune the mesh size of the agarose networks by changing the initial concentration of the agarose solution[4–6]. Supplementary Fig. 6 shows confocal microscopy images of fluorescently labeled agarose gels of varying concentration. To quantify the mesh size, we first azimuthally average the 2D fast Fourier transform (FFT) of the confocal images, yielding the curves in Supplementary Fig. 7. Subsequently, we identify the peak at small $1/(2k)$, where k is the wavenumber. This corresponds to the mesh size (diameter). The peak and peak width are plotted in Supplementary Fig. 8, representing the mesh size and its variation. These mesh sizes are similar to literature values for agarose gels[5–7].

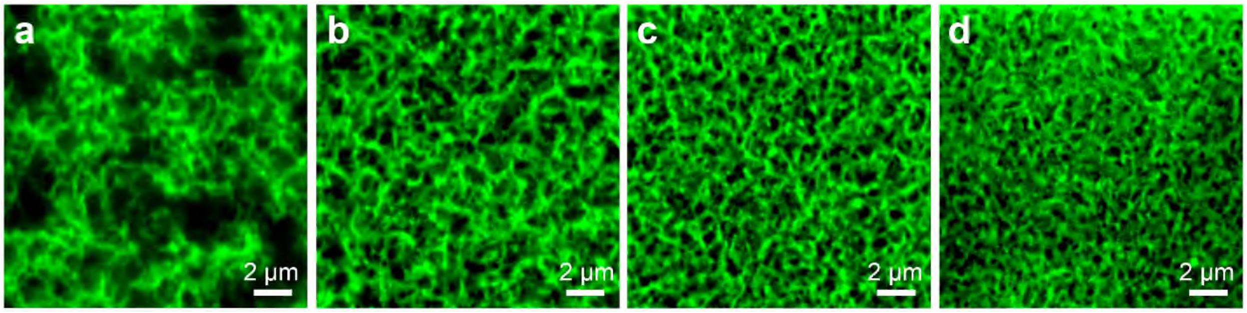

Supplementary Fig. 6: Confocal micrographs of fluorescently-labeled hydrogels with varying agarose concentration: **(a)** 0.3%, **(b)** 0.8%, **(c)** 1.3%, and **(d)** 2.0% w/w.

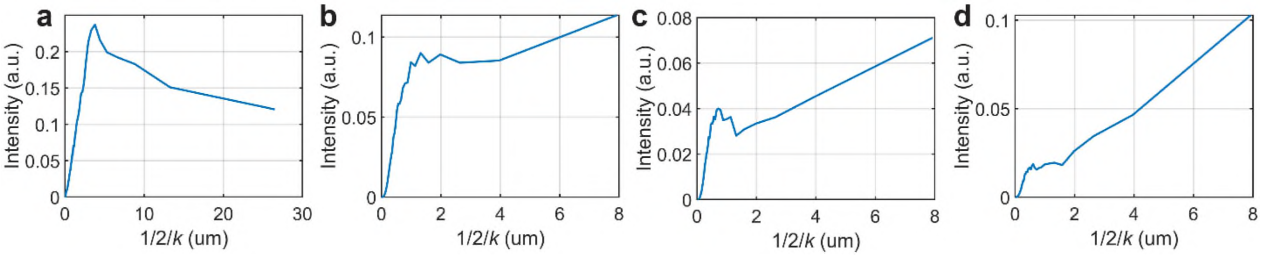

Supplementary Fig. 7: Plots of the intensity profile from azimuthally averaging the 2D fast Fourier transform of the micrographs in Supplementary Fig. 6, for hydrogels with varying agarose concentration: **(a)** 0.3%, **(b)** 0.8%, **(c)** 1.3%, and **(d)** 2.0% w/w. Note that the *x*-axis has been converted to units of length.

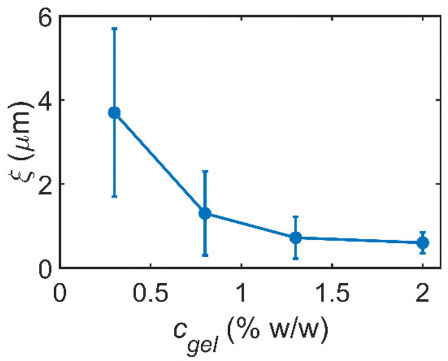

Supplementary Fig. 8: Agarose gel mesh sizes obtained from the peaks of the intensity profiles in Supplementary Fig. 7. Variations in the mesh size are estimated from the widths of the peaks.

Supplementary Note 3: Additional phase separation kinetics data

In Supplementary Fig. 9, we include microscopy time series for condensates growing within agarose gels of different concentrations. We observe that growth occurs via abrupt interface jumps in all gels. As the gel concentration is increased, the structure of condensates becomes smaller and more tortuous as they permeate yet smaller pore spaces.

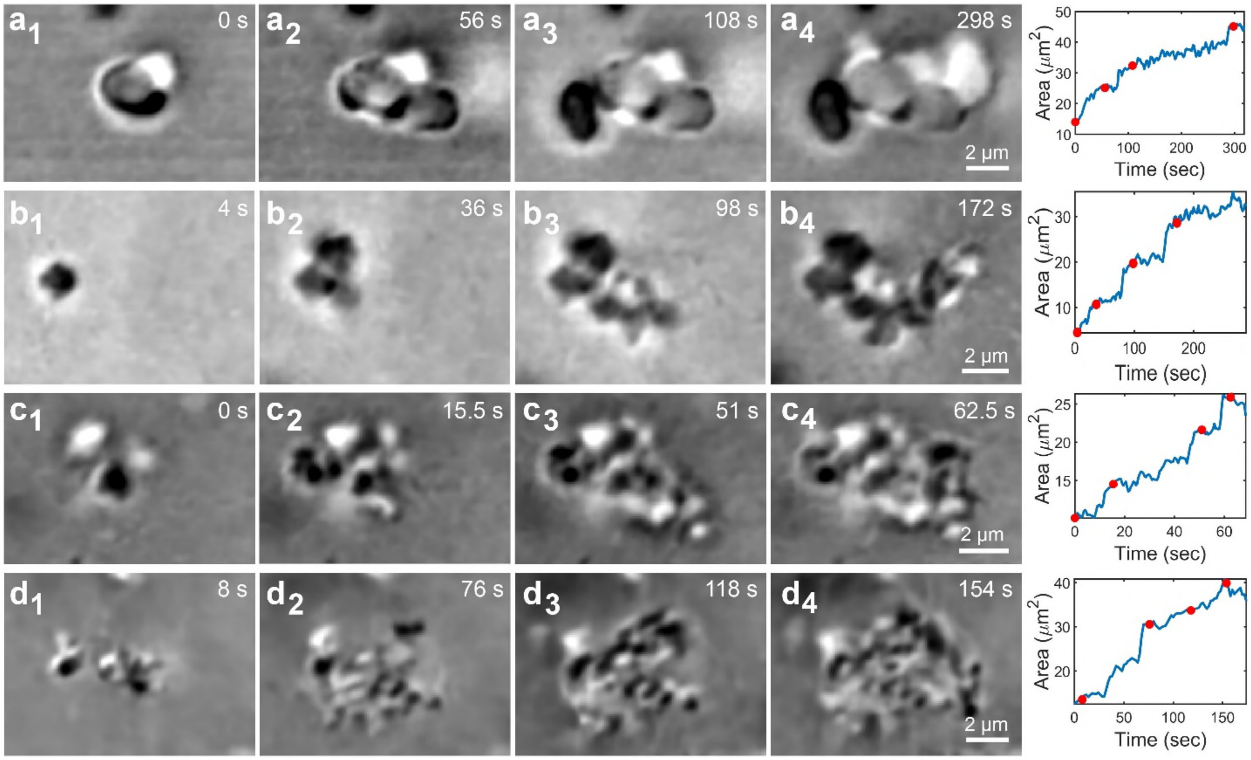

Supplementary Fig. 9: Bright-field microscopy time-series of the condensation of decane in gels of varying agarose concentration: **(a)** 0.3%, **(b)** 0.8%, **(c)** 1.3%, and **(d)** 2.0% w/w. Plots track the evolution of condensate in-plane area vs. time. Red markers indicate the four time points corresponding to the micrographs.

Here, we provide an estimate of the condensation rate of oil from solution. In Supplementary Fig. 10 we show three frames from a bright-field microscopy time series of a decane condensate growing in a 0.3% w/w agarose gel (same experiment as Supplementary Fig. 9a). Due to the large size of the agarose network pore space at this gel concentration (see Supplementary Fig. 6 for example), there are times during which the condensate can grow in the pore space with minimal constraint by the surrounding fibrils. The time series in Supplementary Fig. 10 shows one such time, between $t = 16$ and 28 s, where the condensate grows uniformly in time, rather than via abrupt jumps (also see Supplementary Movie 5).

Growth of the region highlighted by the yellow shape occurs in a 12-second interval. This yellow region has a 2D area of approximately 2.4 μm^2. Approximating the region as the central

slice of a 3D sphere yields a volume of 6.0 μm^3. Formation of this volume in a 12-second interval corresponds to a flow rate of approximately 0.23 fL/s.

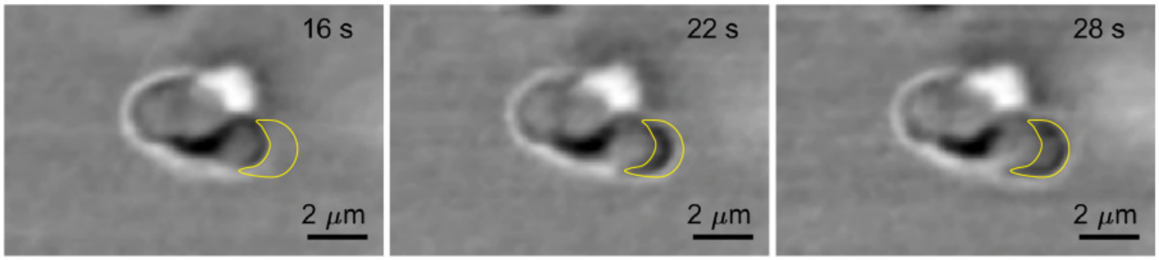

Supplementary Fig. 10: Bright-field microscopy time-series of the condensation of decane a 0.3% w/w agarose gel. Due to the large mesh size, growth of the condensate lobe in the bottom right is unconstrained between $t = 16$ and 28 s, and growth occurs continuously in time, rather than in abrupt jumps (also see Supplementary Movie 5). The yellow shapes identify the region which grows between $t = 16$ and 28 s.

Here, we provide an estimate of the fluid redistribution volume flow rate during a jump of the growing interface in Fig. 2 (and Supplementary Movie 9). In Supplementary Fig. 11, we show fluorescence confocal images from four different time frames. Note that the times shown here in the SI are slightly different from those shown in Fig. 2. Here in the SI, we show $t = 54$, 56, 74, and 76 s, whereas in Fig. 2, we show $t = 54$, **58**, 74, and **78** s. We chose those times for Fig. 2 for visual clarity in the plot of in-plane area (Fig. 2b).

A 2-second interval (the frame exposure time) passes between the first two frames of Supplementary Fig. 11. The region which appears abruptly in that time, outlined in yellow, has a 2D area of approximately 4.0 μm^2. Approximating the region as the central slice of a 3D sphere yields a volume of 6.0 μm^3, corresponding to a flow rate of at least 3.0 fL/s. A similar analysis on the lobe that appears between the 3rd and 4th frames of Supplementary Fig. 11 yields a 2D area of 7.3 μm^2 and corresponding volume of 14.8 μm^3, yielding a flow rate of 7.4 fL/s. These are estimates of a minimum volume flow rate since in reality, fluid redistribution may occur over a substantially shorter period than 2 s.

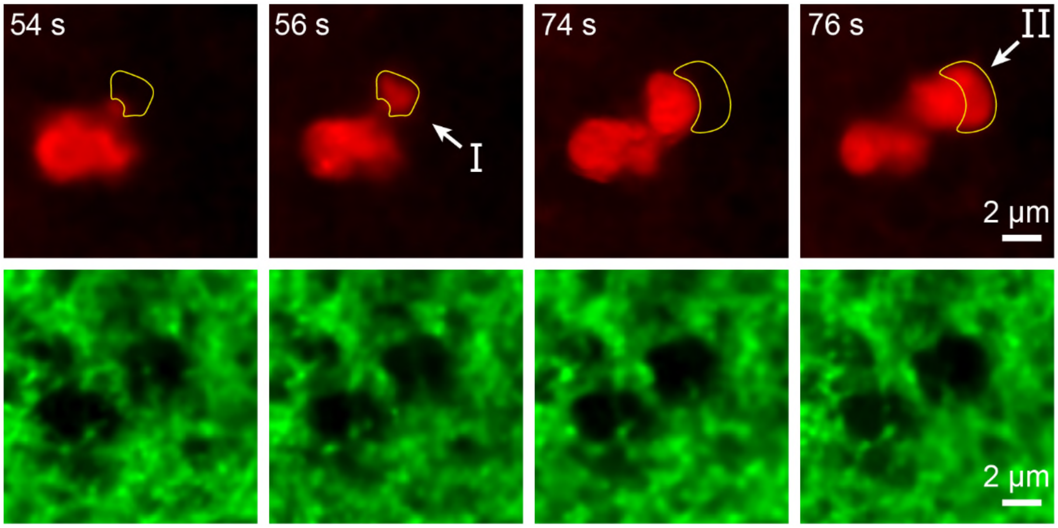

Supplementary Fig. 11: Fluorescence confocal microscopy time series showing abrupt growth jumps of a decane condensate (red) within a 0.3% w/w agarose network (green). The yellow shapes identify regions I and II which appear within 2 s, the exposure time of the sequential images.

Supplementary Note 4: Underwater contact angle measurements

We determine that decane is a non-wetting phase for agarose using underwater contact angle measurements. Underneath a slab of agarose gel which floats on the surface of water, a 31 uL droplet of decane is injected. The measured contact angle between the decane droplet and the agarose is $\theta \approx 180° - 36.2° = 143.8°$ (Supplementary Fig. 12) and thus the decane is highly non-wetting for agarose compared to the aqueous phase.

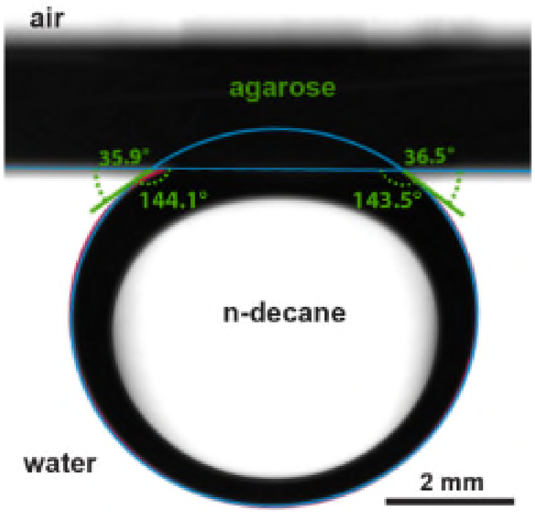

Supplementary Fig. 12: Optical image of a decane droplet contacting an agarose gel, underwater. Underwater contact angle measurements yield a contact angle of 144.1°. The droplet volume is 31 uL and the measurement was performed at 23° C.

Supplementary Note 5: Interface jump size distribution data and *Ca* estimation

Here, we calculate the probability distribution of interface jump sizes during oil condensation. We perform this analysis on bright-field microscopy time series of the condensation process in gels of varying concentration. First, we take the difference between video frames separated by a time increment of 4 seconds. Next, we binarize these difference images and segment the objects, with these objects corresponding to interface jumps that occurred between the subsequent times. Finally, we bin and plot these jumps according to their lateral area (Supplementary Fig. 13a). In invasion percolation of a non-wetting phase into a porous medium, a power law distribution of jumps sizes is expected[8,9], and power laws fit the obtained probability distributions reasonably well. We note that substantial error may be incurred due to poor optical resolution of small jumps, a low signal to noise ratio, and insufficient jump statistics.

In Supplementary Fig. 13b we plot the number of jumps that occur as a function of time. We see that the number of observed jumps rises sharply as condensation initiates and falls to near zero within several minutes as solute is depleted from solution.

Here, we calculate the viscosity ratio and capillary number associated with the observed interface jumps and show that they are within the expected regime for growth via abrupt bursts[10,11]. The wetting phase is water and the non-wetting phase is decane. The viscosity of the wetting phase is η_w = 1.002 cP,[12] and the viscosity of the non-wetting phase is η_{nw} = 0.87 cP.[13] The viscosity ratio is $M = \eta_{nw}/\eta_w$ = 0.86. We fit lines to the lateral area vs. time curves in Supplementary Fig. 9 to obtain an average linear growth velocity of v_{growth} = 0.01 um/s. We estimate the oil–water interfacial tension to be $\gamma_{ow} \approx$ 8 mN/m during condensation (see Supplementary Fig. 4). This yields a capillary number of $Ca = \eta_{nw} v_{growth}/\gamma_{ow}$ = 1.3 × 10^{-9}. This is well within the regime expected for growth via abrupt bursts, at $Ca \ll 1$.[14–16]

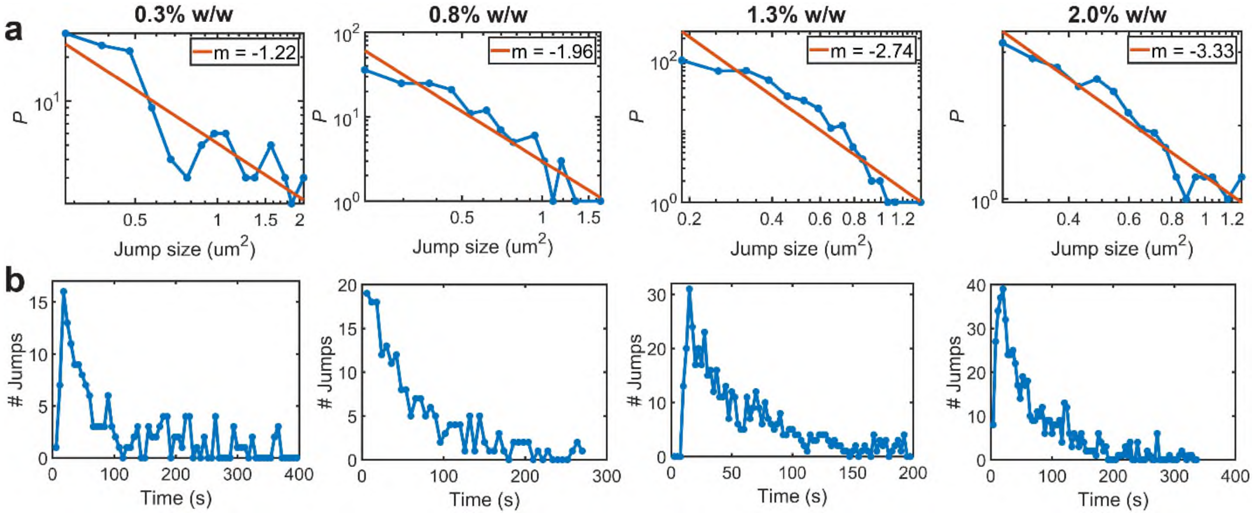

Supplementary Fig. 13: (a) Probability distributions of interface jump lateral size for various gel concentrations from 0.3% to 2.0% w/w. The red line is a power law fit, with fitted exponent *m*. **(b)** Number of observed jumps plotted as a function of time.

Supplementary Note 6: Fibril tensile strength estimation

Here, we estimate the stress applied to the restraining fibril in Fig. 3b3 at the point of yielding. Yielding and elongation of the last restraining element is observed to initiate at $t \approx$ 600 s (inset of Fig. 3c). We consider the restraining element visible in Fig. 3b3 to form a hoop with radius R and cross-sectional radius r_f which constrains the condensate in Fig. 3a3 around its waist (schematic in Supplementary Fig. 14). In this geometry, the net force acting on the hoop in the outward normal direction is

$$F_n = 2(2\pi R)\, \gamma_{ow} \cos{(\phi)}$$

where ϕ is the contact angle indicated in Supplementary Fig. 14. Thus, within the hoop, the azimuthal tension, F_a, satisfies

$$F_a\, d\theta = 4\pi\gamma_{ow} \cos(\phi)\, ds$$

where

$$d\theta = \frac{ds}{R}$$

This yields

$$F_a = 4\pi R\, \gamma_{ow} \cos(\phi)$$

which is the longitudinal tension within the fibril. The corresponding tensile stress is

$$\sigma = \frac{F_a}{\pi r_f^2} = \frac{4\pi R\, \gamma_{ow} \cos(\phi)}{\pi r_f^2}$$

From SEM images in Supplementary Fig. 22, the agarose fibril radii are approximately $r_f \approx$ 10 nm, which matches literature results[4,17]. From Fig. 3a3, the contact angle is approximately $\phi \approx$ 60°. The hoop radius is $R \approx 1\ \mu$m. We use the critical interfacial tension at which network fracture is observed, γ_c = 17 mN/m (for c_{gel} = 0.8% w/w). With these values, estimate a fibril yield stress of

$$\sigma \approx\ 340 \text{ MPa}$$

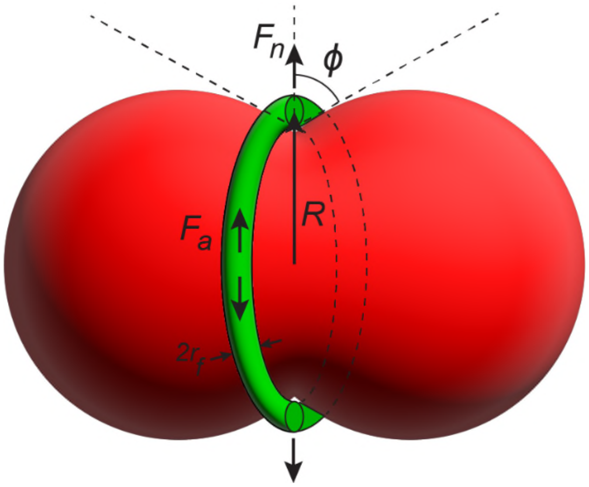

Supplementary Fig. 14: Schematic of the geometry used to estimate the fibril yield stress. The red object is the decane condensate and the green hoop is the restraining agarose fibril. F_n is the normal force acting in the outward direction. ϕ is the local contact angle between the hoop and the condensate. R is the diameter of the condensate at its waist. F_a is the azimuthal tension that acts within the hoop. r_f is the radius of the fibril.

Supplementary Note 7: Decane dissolution experiments

To assess the elasticity and plasticity of network deformation, we soak gels in an excess of ethanol to dissolve away the condensed oil droplets, as described in the Materials and Methods section. We present this data in Supplementary Fig. 15 to Supplementary Fig. 18. In these figures, the 1st and 3rd columns are microscopy images of the condensates and network prior to oil dissolution, while the 2nd and 4th columns are images after dissolution.

The 1st and 2nd columns correspond to experiments performed in the absence of surfactant, while the 3rd and 4th columns correspond to experiments performed with an excess of surfactant. In surfactant experiments, we use the anionic surfactant laureth-4 (L4), with a surfactant-oil-ratio of 1:20 (~5% v/v). At this concentration, the interfacial tension between the oil-rich and water-rich phases is 0.5 mN/m as measured by pendant droplet tensiometry, and network fracture is completely precluded. The reason we employ L4 (HLB = 9.7)[18] in these dissolution experiments is to avoid micelle-induced solubilization of oil phase itself by TX-100 (HLB = 13.5)[19], which occurs during the long time necessary for solvent exchange and dissolution to occur.

Upon removal of oil droplets, we find that the deformation is largely plastic, with the network cavities generally maintaining their shape. However, some shrinkage of the cavity is observed, implying that the network response is at least partially elastic. We find that cavities reduce their area by between 8.8% to 21% after droplet dissolution, with specific values provided in the captions of Supplementary Fig. 15 to Supplementary Fig. 18.

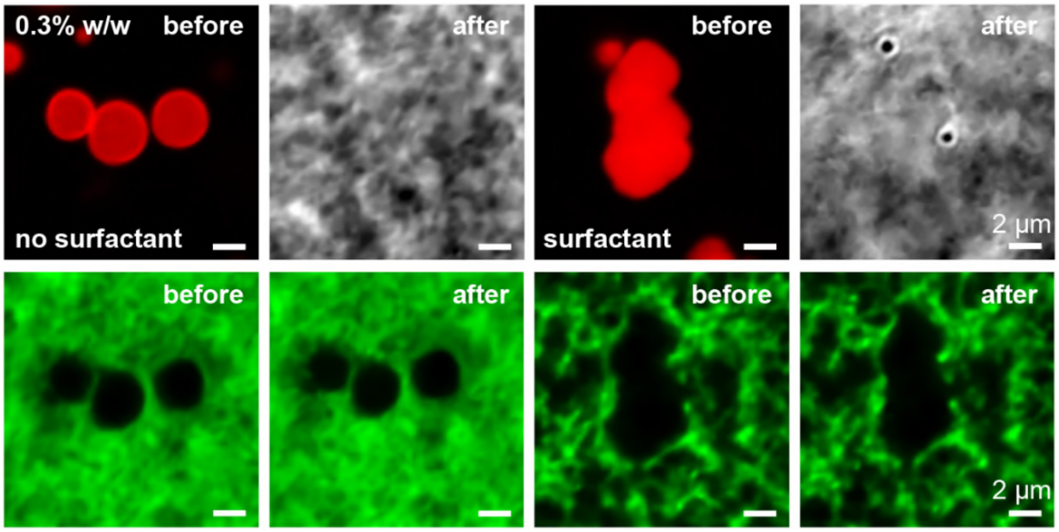

Supplementary Fig. 15: Dissolution experiments in 0.3% w/w gels with (columns 1 and 2) and without (columns 3 and 4) L4 surfactant. In the case without surfactant, the center cavity shrinks from 7.945 um^2 to 6.242 um^2 upon oil dissolution, a 21% reduction. In the case with surfactant, the cavity shrinks from 29.39 um^2 to 26.59 um^2, a 9.5% reduction.

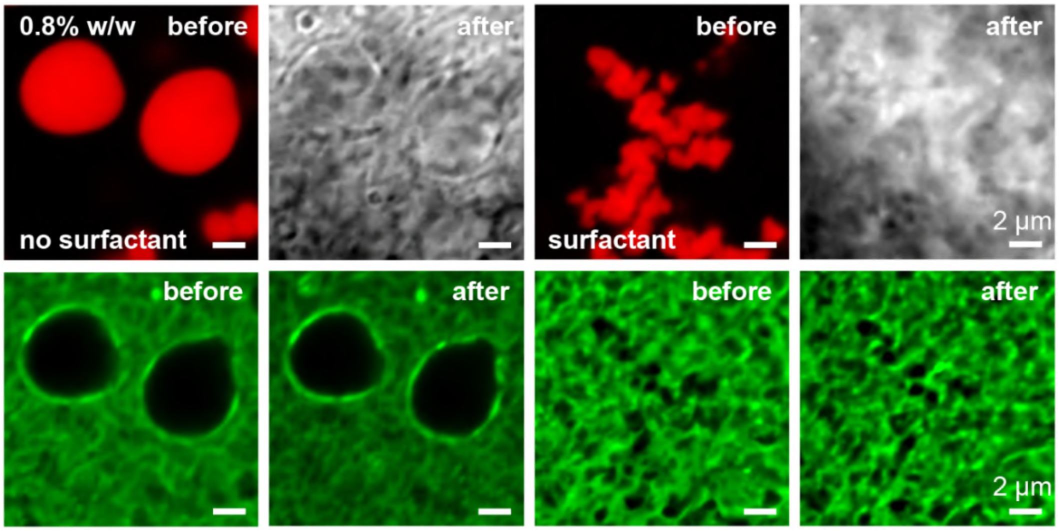

Supplementary Fig. 16: Dissolution experiments in 0.8% w/w gels with (columns 1 and 2) and without (columns 3 and 4) L4 surfactant. In the case without surfactant, the left cavity shrinks from 23.18 um^2 to 21.12 um^2 upon oil dissolution, an 8.8% reduction. In the case with surfactant, no cavities beyond the mesh size are observed. However, deformation of the network at the mesh-scale is still present after oil dissolution.

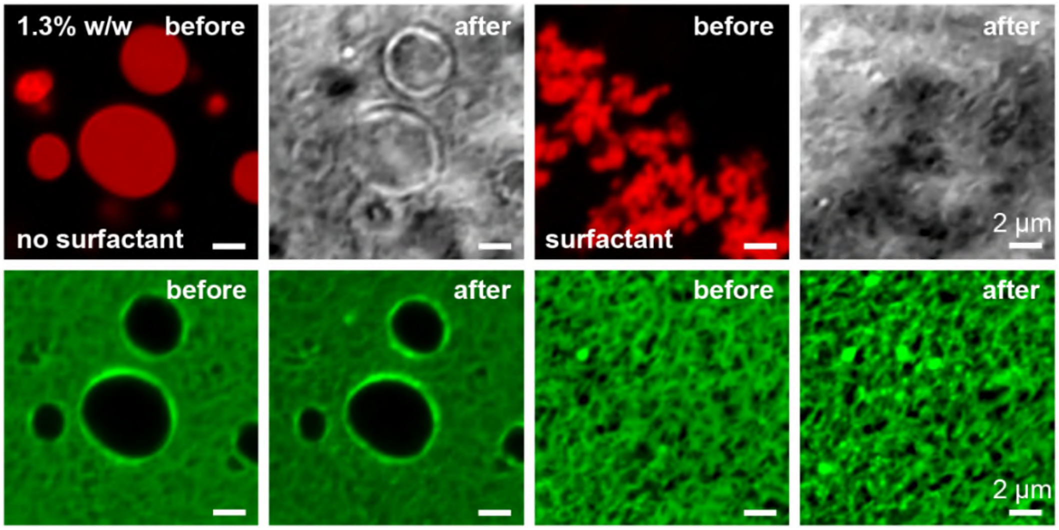

Supplementary Fig. 17: Dissolution experiments in 1.3% w/w gels with (columns 1 and 2) and without (columns 3 and 4) L4 surfactant. In the case without surfactant, the left cavity shrinks from 18.11 um^2 to 16.47 um^2 upon oil dissolution, a 11% reduction. In the case with surfactant, no cavities beyond the mesh size are observed. However, deformation of the network at the mesh-scale is still present after oil dissolution.

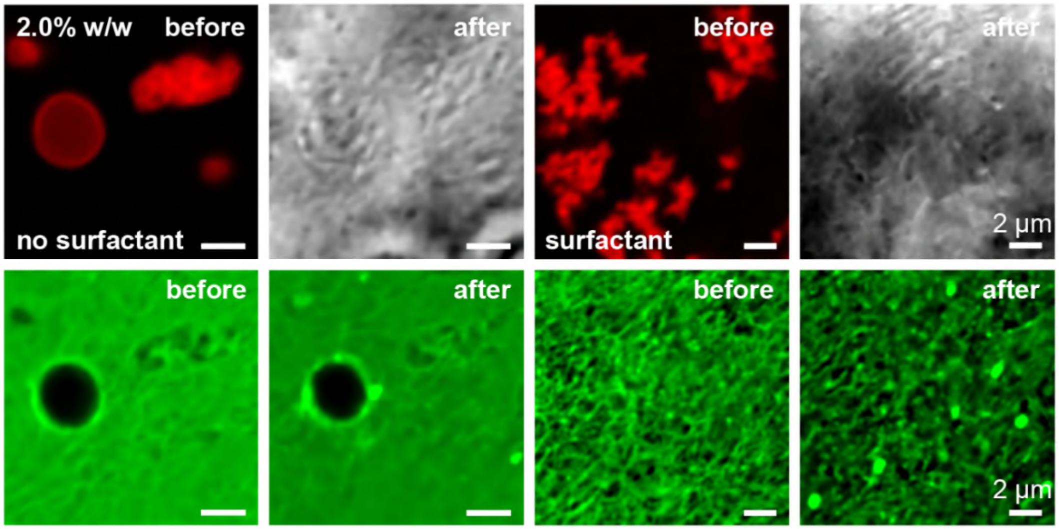

Supplementary Fig. 18: Dissolution experiments in 2.0% w/w gels with (columns 1 and 2) and without (columns 3 and 4) L4 surfactant. In the case without surfactant, the left cavity shrinks from 5.3 um^2 to 4.3 um^2 upon oil dissolution, a 18% reduction. In the case with surfactant, no cavities beyond the mesh size are observed. However, deformation of the network at the mesh-scale is still present after oil dissolution.

Supplementary Note 8: Additional surfactant experiments

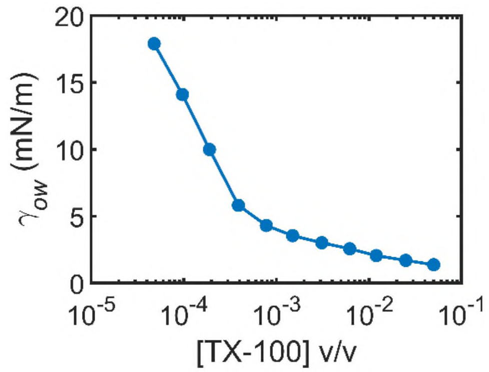

Supplementary Fig. 19: Interfacial tension between decane and water, γ_{ow}, with varying TX-100 concentration in the aqueous phase. Interfacial tension is measured with pendant droplet tensiometry.

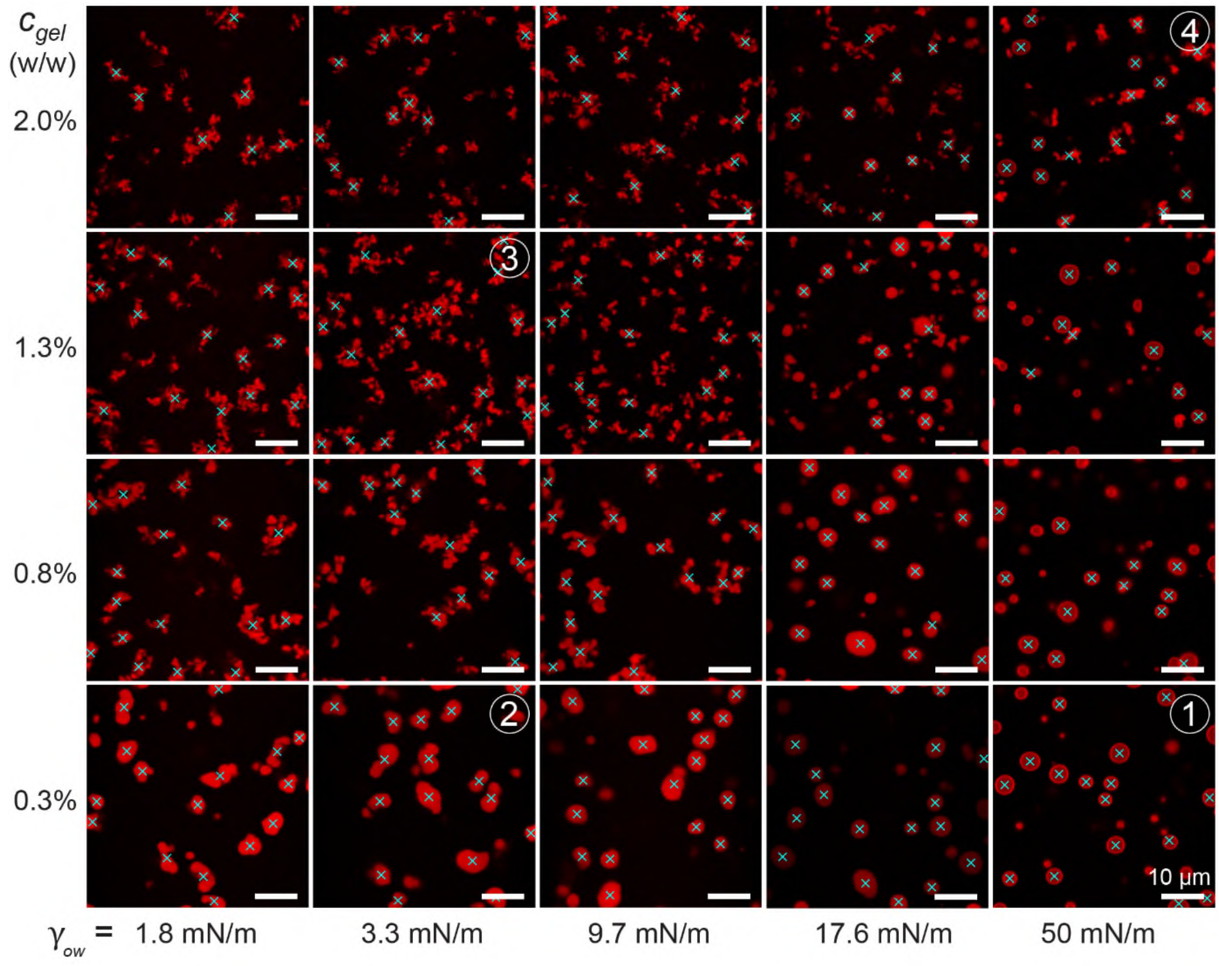

Supplementary Fig. 20: Fluorescence confocal micrographs of condensates in gels with varying gel concentration, c_{gel}, and oil–water interfacial tension, γ_{ow}. Cyan crosses indicate segmented

objects with area greater than 4 μm^2 whose lateral sizes are used in the histograms in Supplementary Fig. 21 below. All scale bars are 10 μm. The circled numbers ①–④ correspond to the images shown in Fig. 6 of the main text. Note that these images are the full field-of-view images used to generate the histograms in Supplementary Fig. 21 below. The images in Fig. 6 of the main text show a reduced field-of-view for clarity.

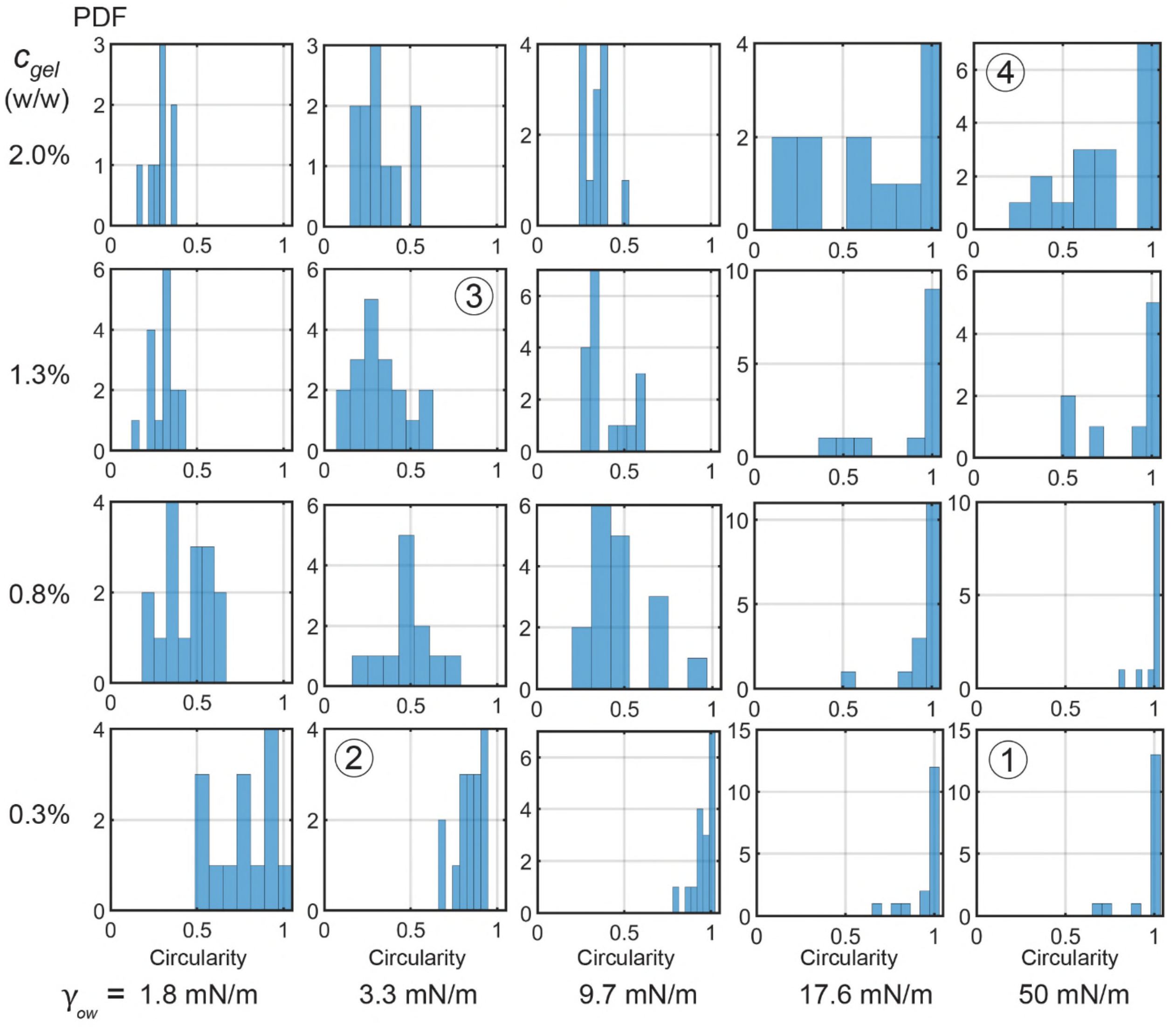

Supplementary Fig. 21: Histograms of condensate circularity in gels with varying c_{gel} and γ_{ow}. The segmented objects used to generate the histograms are indicated with cyan crosses in Supplementary Fig. 20 above. The circled numbers ①–④ correspond to the histograms shown in Fig. 6 of the main text.

Supplementary Note 9: Cross-sectional SEM data

Here, we present additional SEM images of the network structure after lyophilization to remove water and decane and fracture to reveal the cross section. In Supplementary Fig. 22a we show electron micrographs of the deformed networks for four different gel agarose concentrations, 0.3%, 0.8%, 1.3%, and 2.0% w/w. Qualitatively, the shell density increases with increasing gel concentration, corroborating confocal microscopy images (columns 1 and 2 of Supplementary Fig. 15 to Supplementary Fig. 18). In Supplementary Fig. 22b we show electron micrographs of the native gel structure, where a variety of mesh sizes are observed.

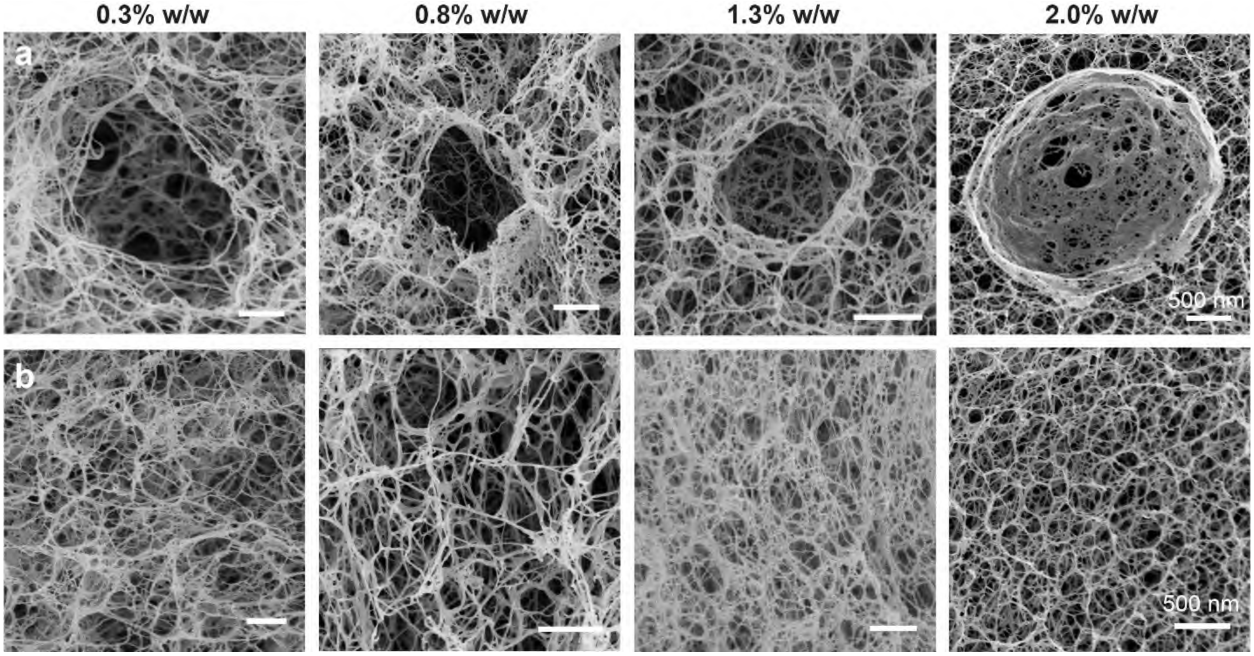

Supplementary Fig. 22: (a) Cross-sectional SEM images of the agarose gels after lyophilization and fracture to reveal the structure of the densified shell, for four different gel concentrations. **(b)** SEM images of the undeformed network structure.

Supplementary References

1. Skrzecz, A., Shaw, D., Maczynski, A. & Skrzecz, A. IUPAC-NIST solubility data series 69. Ternary alcohol-hydrocarbon-water systems. *J Phys Chem Ref Data* **28**, 983–992 (1999).
2. You, J. B., Lohse, D. & Zhang, X. Tuning Composition of Multicomponent Surface Nanodroplets in a Continuous Flow-In System. *Adv Mater Interfaces* **8**, (2021).
3. Callister, W. & Rethwisch, D. *Fundamentals of Materials Science and Engineering*. (John Wiley & Sons, 2015).
4. Bertula, K. *et al.* Strain-Stiffening of Agarose Gels. *ACS Macro Lett* **8**, 670–675 (2019).
5. Moore, M. J. *et al.* The dance of the nanobubbles: Detecting acoustic backscatter from sub-micron bubbles using ultra-high frequency acoustic microscopy. *Nanoscale* **12**, 21420–21428 (2020).
6. Pernodet, N., Maaloum, M. & Tinland, B. Pore size of agarose gels by atomic force microscopy. *Electrophoresis* **18**, 55–58 (1997).
7. Narayanan, J., Xiong, J. Y. & Liu, X. Y. Determination of agarose gel pore size: Absorbance measurements vis a vis other techniques. *J Phys Conf Ser* **28**, 83–86 (2006).
8. Xu, L., Davies, S., Schofield, A. B. & Weitz, D. A. Dynamics of drying in 3D porous media. *Phys Rev Lett* **101**, 29–32 (2008).
9. Martys, N., Robbins, M. O. & Cieplak, M. Scaling relations for interface motion through disordered media: Application to two-dimensional fluid invasion. *Phys Rev B* **44**, 12294–12306 (1991).
10. Berg, S. *et al.* Real-time 3D imaging of Haines jumps in porous media flow. *Proc Natl Acad Sci U S A* **110**, 3755–3759 (2013).
11. Datta, S. S., Ramakrishnan, T. S. & Weitz, D. A. Mobilization of a trapped non-wetting fluid from a three-dimensional porous medium. *Physics of Fluids* **26**, (2014).
12. Korson, L., Drost-Hansen, W. & Millero, F. J. Viscosity of water at various temperatures. *Journal of Physical Chemistry* **73**, 34–39 (1969).
13. Estrada-Baltazar, A., Iglesias-Silva, G. A. & Barrufet, M. A. Liquid viscosities of pentane and pentane + decane from 298.15 K to 373.15 K and up to 25 MPa. *J Chem Eng Data* **43**, 601–604 (1998).
14. An, S., Erfani, H., Godinez-Brizuela, O. E. & Niasar, V. Transition From Viscous Fingering to Capillary Fingering: Application of GPU-Based Fully Implicit Dynamic Pore Network Modeling. *Water Resour Res* **56**, (2020).
15. Lu, N. B., Browne, C. A., Amchin, D. B., Nunes, J. K. & Datta, S. S. Controlling capillary fingering using pore size gradients in disordered media. *Phys Rev Fluids* **4**, 1–12 (2019).
16. Zacharoudiou, I., Boek, E. S. & Crawshaw, J. The impact of drainage displacement patterns and Haines jumps on CO2 storage efficiency. *Sci Rep* **8**, 1–13 (2018).
17. Martikainen, L., Bertula, K., Turunen, M. & Ikkala, O. Strain stiffening and negative normal force of agarose hydrogels. *Macromolecules* **53**, 9983–9992 (2020).
18. Singh, S. *et al.* Mild Oxidation of Thiofunctional Polymers to Cytocompatible and Stimuli-Sensitive Hydrogels and Nanogels. *Macromol Biosci* **13**, 470–482 (2013).
19. Egan, R. W., Jones, M. A. & Lehninger, A. L. Hydrophile lipophile balance and critical micelle concentration as key factors influencing surfactant disruption of mitochondrial membranes. *Journal of Biological Chemistry* **251**, 4442–4447 (1976).
