# Supplementary material for: Liquid–liquid phase separation within fibrillar networks
Source: Nat Commun. 2023 Sep 29;14:6085. doi: 10.1038/s41467-023-41528-8 (PMC10539382; doi:10.1038/s41467-023-41528-8)
Supplement: Supplementary file 3 — Description of Additional Supplementary Files [file 41467_2023_41528_MOESM3_ESM.pdf]

Description of Additional Supplementary Files

Supplementary Movie 1: (Corresponds to Fig. 1b) Bright-field and fluorescence confocal microscopy movie of a decane condensate (red channel) growing via abrupt jumps within a 0.8% w/w agarose gel (green channel). (Note the video time runs from t = -30 to 300 s)

Supplementary Movie 2: (Corresponds to Fig. 1b) Bright-field and fluorescence confocal microscopy movie of the same decane condensate in Movie 1, at later times. Interfacial tension rises as solvent exchange proceeds, and network deformation and fracture can be seen in this video. Movie 2 is played back at 6.66x the speed of Movie 1. (Note the video time runs from t = 300 to 2580 s)

Supplementary Movie 3: Full field-of-view microscopy movie from which Movies 1 is taken. (Note that the video time runs from t = -30 to 560 s) Movie 4: Full field-of-view microscopy movie from which Movies 2 is taken.

Supplementary Movie 4 is played back at 6.66x the speed of Movie 3. (Note that the video time runs from t = 560 to 2580 s)

Supplementary Movie 5: Optical microscopy movie of a decane condensate (bright-field) growing within a 0.3% w/w agarose gel.

Supplementary Movie 6: Optical microscopy movie of a decane condensate (bright-field) growing within a 0.8% w/w agarose.

Supplementary Movie 7: Optical microscopy movie of a decane condensate (bright-field) growing within a 1.3% w/w agarose.

Supplementary Movie 8: Optical microscopy movie of a decane condensate (bright-field) growing within a 2.0% w/w agarose.

Supplementary Movie 9: (Corresponds to Fig. 2a) Fluorescent confocal microscopy movie of a decane condensate (red channel) growing via abrupt jumps within a 0.3% w/w agarose gel (green channel).

Supplementary Movie 10: (Corresponds to Fig. 3a,b) Fluorescent confocal microscopy movie of a decane condensate (red channel) within a 0.8% w/w agarose network (green channel) during the fracture of the agarose network.

Supplementary Movie 11: Fluorescent confocal microscopy movie of a decane condensate (red channel) within a 0.3% w/w agarose network (green channel) during the fracture of the agarose network.

Supplementary Movie 12: (Corresponds to Fig. 5a) Fluorescent confocal microscopy video of the dissolution of a decane condensate (bright-field channel) within a 0.3% w/w agarose gel (green channel).
